# Silver Nanoparticle-Based Assay for the Detection of Immunoglobulin Free Light Chains

**DOI:** 10.3390/ma12182981

**Published:** 2019-09-15

**Authors:** Anna Lizoń, Magdalena Wytrwal-Sarna, Marta Gajewska, Ryszard Drożdż

**Affiliations:** 1 Department of Medical Diagnostics, Faculty of Farmacy, Jagiellonian University Collegium Medicum, Medyczna 9, 30-688 Kraków, Poland; ryszard.drozdz@uj.edu.pl; 2 Academic Centre for Materials and Nanotechnology, University of Science and Technology, 30 Kawiory, 30-055 Kraków, Poland; wytrwal@agh.edu.pl (M.W.-S.); marta.gajewska@agh.edu.pl (M.G.)

**Keywords:** multiple myeloma, amyloidosis, laser light scattering, silver nanoparticles

## Abstract

There is a wide spectrum of malignant diseases that are connected with the clonal proliferation of plasma cells, which cause the production of complete immunoglobulins or their fragments (heavy or light immunoglobulin chains). These proteins may accumulate in tissues, leading to end organ damage. The quantitative determination of immunoglobulin free light chains (FLCs) is considered to be the gold standard in the detection and treatment of multiple myeloma (MM) and amyloid light-chain (AL) amyloidosis. In this study, a silver nanoparticle-based diagnostic tool for the quantitation of FLCs is presented. The optimal test conditions were achieved when a metal nanoparticle (MNP) was covered with 10 particles of an antibody and conjugated by 5–50 protein antigen particles (FLCs). The formation of the second antigen protein corona was accompanied by noticeable changes in the surface plasmon resonance spectra of the silver nanoparticles (AgNPs), which coincided with an increase of the hydrodynamic diameter and increase in the zeta potential, as demonstrated by dynamic light scattering (DLS). A decrease of repulsion forces and the formation of antigen–antibody bridges resulted in the agglutination of AgNPs, as demonstrated by transmission electron microscopy and the direct formation of AgNP aggregates. Antigen-conjugated AgNPs clusters were also found by direct observation using green laser light scattering. The parameters of the specific immunochemical aggregation process consistent with the sizes of AgNPs and the protein particles that coat them were confirmed by four physical methods, yielding complementary data concerning a clinically useful AgNPs aggregation test.

## 1. Introduction

Multiple myeloma, the second most common hematologic malignancy, is characterized by the clonal proliferation of plasma cells, their prolonged survival, and the accumulation of clonal plasma cells in bone marrow. It is accompanied by the presence of monoclonal immunoglobulin and immunoglobulin free light chains (FLCs) in the serum, urine, or both. FLCs or their deposits may accumulate in tissues, leading to end organ damage. The most common clinical manifestations of symptomatic multiple myeloma are anemia, infections, lytic or osteopenic bone disease, and renal failure [1,2]. Another disease associated with immunoglobulin free light chains is amyloidosis. It is caused by an aggregation of misfolded FLCs or their fragments in vital organs (the kidneys, heart, liver, or peripheral nerves). Deposits of amyloid fibrils lead to an impaired function of affected organs. There is a common consensus that amyloidosis is underdiagnosed. Without an accurate diagnosis and proper treatment, the typical survival period of patients with undiagnosed amyloidosis and cardiac involvement is estimated to be about six months, so a fast diagnosis is crucial. The treatments currently available significantly increase survival [3,4].

From the discovery of the Bence‒Jones protein, which was later found to be an immunoglobulin free light chain, a new era in the diagnosis and monitoring of MM and related disorders was ushered in. The quantitative determination of free light chains is considered to be the gold standard in the detection and treatment of multiple myeloma and AL amyloidosis [2,5,6].

Metal nanoparticles (MNPs) are widely used in many fields of medicine, such as diagnostics, therapy, and medical imaging. Silver and gold nanoparticles have attracted a lot of attention due to their unique optical properties, resulting from localized surface plasmon resonance (LSPR) [7,8]. LSRP depends on several factors, such as the size and shape of particles and the distance between them. When the distance between nanoparticles decreases, or there are changes in the dielectric constant of the local environment on the surfaces of MNPs (changes in the nanoparticle environment or particle agglomeration), a change in the absorbance spectrum occurs, which is accompanied by a change in the colloid color [9].

One of the promising uses of MNPs, based on their unique physical properties, is in the development of laboratory assays for detecting different analytes. There are many potential mechanisms for the detection of the biological markers of diseases. One of the mechanisms is the immunochemical interaction between biomolecules of interest and MNPs, which is captured using antibodies. Antibody–antigen interactions are based on biospecific recognition, which has a high selectivity [10,11]. Antibody‒antigen interaction can either cause direct agglutination [12,13] or inhibit aggregation caused by another destabilizing factor affecting the optical properties of nanoparticles [14,15].

Nanoparticle-based assays are considered a promising alternative to classic latex assays. Silver and gold nanoparticles (NPs) have a remarkably high absorption coefficient and strongly distance-dependent optical properties. The interaction of antibodies immobilized on MNP surfaces with their antigens causes nanoparticle aggregation and an LSPR shift, which is indicated by a change in a solution’s color [11,16].

While there are many theoretical papers concerning silver nanoparticles’ properties and their possible use in diagnostics, there are still few practical laboratory assays, and the proposed solutions differ significantly. 

In this pilot study, a diagnostic tool for the detection of immunoglobulin free light chains in urine, based on silver nanoparticles, is presented. E and optimization of the molar ratio of the metal nanoparticles, antibodies, and antigen at which the optical changes occur were performed.

## 2. Materials and Methods 

Anti-lambda bound and free polyclonal antibodies (SCE239M) were obtained from Biameditek sp. z o.o. (Białystok, Poland). Free light chain lyophilizate was obtained from urine samples of patients diagnosed with monoclonal gammopathy and Bence‒Jones proteinuria. To isolate the protein (FLCs), the salting out method using an ammonium sulfate solution was used. The use of human biological material for the research was approved by the Bioethics Committee of the Jagiellonian University: KBET/161/B/2009 and 1072.6120.248.2017 (25.10.2018). AgNO_3_, NaBH_4_, trisodium citrate, and all other chemical reagents were purchased from Sigma-Aldrich Co. (St. Louis, MO, USA).

### 2.1. Synthesis of Silver Nanoparticles

AgNPs were prepared according to the method reported by Creighton [17]. This method is based on the reduction of silver nitrate using sodium borohydride. Briefly: 0.5 mL of an aqueous solution of AgNO_3_ (0.01 M) and 0.5 mL trisodium citrate (0.01 M) were added to 19 mL of chilled distilled H_2_O. Then, a freshly prepared aqueous solution of NaBH_4_ (0.6 mL, 0.01 M) was added drop-by-drop under vigorous stirring at 0 °C. After NaBH_4_ was added, the vigorous magnetic stirring was maintained for 3 min. The resulting solution changed to a stable light yellow color, indicating the formation of Ag nanoparticles. The stable silver colloid solution was stored at 4 °C until use.

### 2.2. Functionalization of AgNPs with Antibodies

The silver nanoparticles were coated with antibodies by adding 1 μL of the anti-FLCs antiserum to 10 mL of the AgNPs solution. After stirring well, the mixture was incubated for 30 min at 37 °C.

### 2.3. The Detection of FLCs by the AgNPs-ab Probe

In order to follow the immunological reaction between antibody-functionalized AgNPs and the target proteins, immunoglobulin free light chains (FLCs), a stock PBS solution, at a concentration of 2.2 g/L, was prepared. 

The preparation of the primary ab-functionalized AgNPs and antigen system was as follows: working solutions were obtained by mixing the antibody-functionalized nanoparticles (1 mL) and different volumes of the stock FLCs solution (1, 2, 3, 4, 5, 7 and 10 μL). The final FLCs concentrations were 2.2–22 mg/L (100–1000 Nm). To obtain lower FLCs concentrations (0.2–1.1 mg/L, 10 nM–50 nM), appropriate dilutions (10×, 8×, 5×, 4×, 3×, 2×) of the stock solution were added to 1 mL of antibody-coated silver nanoparticles. After stirring them well, the mixtures were incubated at 37 °C.

### 2.4. The Detection of FLCs by the AgNPs-ab Probe

#### 2.4.1. UV/Vis Spectroscopy

Citrate-stabilized AgNPs, antibody-modified AgNPs and AgNPs + ab + FLCs aggregates were analyzed by UV/Vis spectroscopy (MultiscanSky, Thermo Scientific, Waltham, MA, USA) within a range of 350–600 nm. Path length adjustment to 1 cm was performed.

#### 2.4.2. Transmission Electron Microscopy

Transmission electron microscopy (TEM) observations were carried out on a FEI Tecnai TF20 X-TWIN (FEG) microscope (Thermo Scientific, Waltham, MA, USA), working at an accelerating voltage of 200 kV. Samples for TEM observations were prepared by drop-casting on carbon-coated copper TEM grids. DeltaOptical DLT CamViewer software (ver. x64, 3.7.12277.20180703) was used for TEM image analysis. Diameter values obtained by the distance selection tool previously calibrated to the scale bar imprinted on the TEM images were determined. 

#### 2.4.3. Dynamic Light Scattering 

A Malvern Nano ZS light scattering apparatus (Malvern Instruments Ltd., Malvern, UK) was used for dynamic light scattering (DLS) measurements. The time-dependent autocorrelation function of the photocurrent was acquired every 10 s, with 15 acquisitions for each run. The samples were illuminated by a 633 nm laser, and the intensity of the light, scattered at an angle of 173°, was measured by an avalanche photodiode. The ζ-averaged hydrodynamic mean diameters (dz), polydispersity (PDI), and distribution profiles of the samples were calculated using the software provided by the manufacturer. The following samples were measured: 1) citrate-stabilized AgNPs, 2) AgNPs coated with an antibody, 3) AgNPs coated with an antibody and incubated with FLCs (1 mL:3 µL). The third sample was measured after 30 min, 1 h, and 2 h of incubation at 37 °C, with gentle and continuous stirring.

#### 2.4.4. Laser Light Scattering 

**Figure 1:**
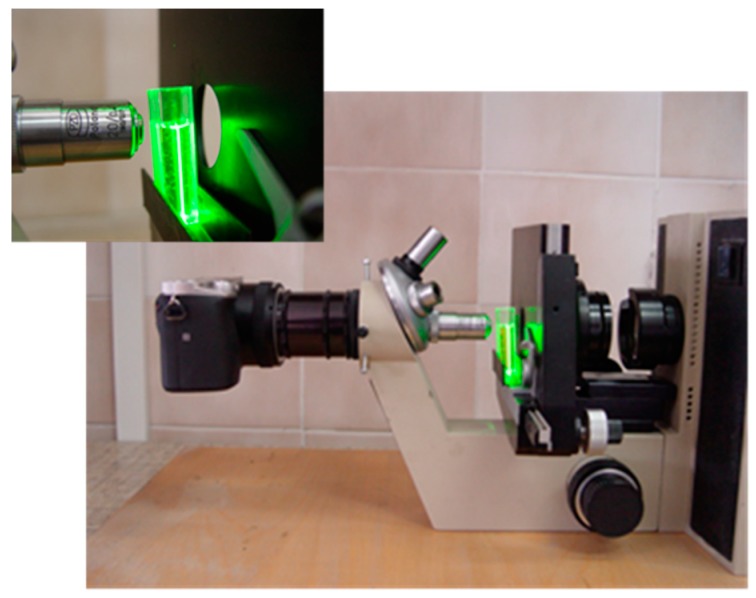
Self-made measurement system for the observation of laser light scattering.

Observations of the laser light scattering patterns of the tested NPs solutions were performed using a self-made measurement system. The system (Figure 1) consisted of:

(1) A green semiconductor laser of 532 nm, which was situated above the sample;
(2) A optical microscope placed horizontally, with 40× magnification and a long working distance objective lens placed in the revolver;
(3) Samples were placed in standard plastic cuvettes (1 cm pathlength); and
(4) Scattering patterns were recorded using a SONY alpha 6000 camera (Minato, Tokio, Japan).

## 3. Results and Discussion

### 3.1. Characteristics of Silver Nanoparticles 

**Figure 2:**
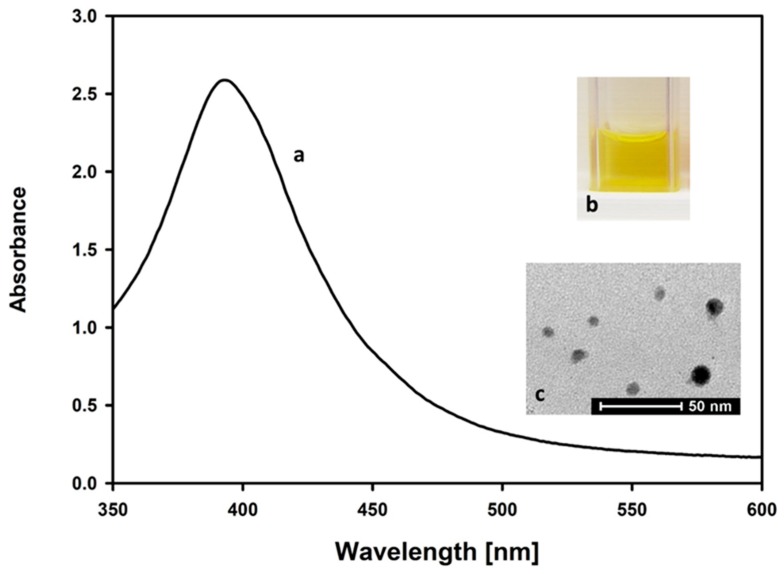
Silver nanoparticles prepared by a chemical reduction of silver nitrate (V) using sodium borohydride: (**a**) characteristic UV-Vis spectrum, with the maximum absorbance at 395 nm, (**b**) color of the solution, (**c**) transmission electron microscope image of the synthesized AgNPs.

**Figure 3:**
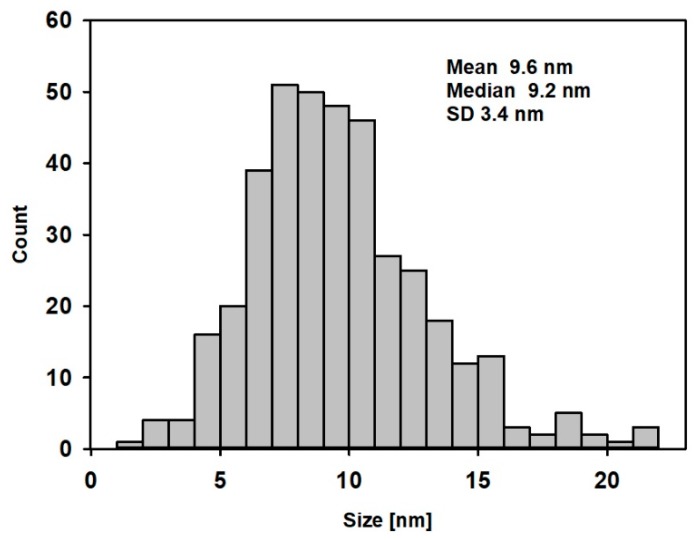
TEM particle size distribution of AgNPs.

**Figure 4:**
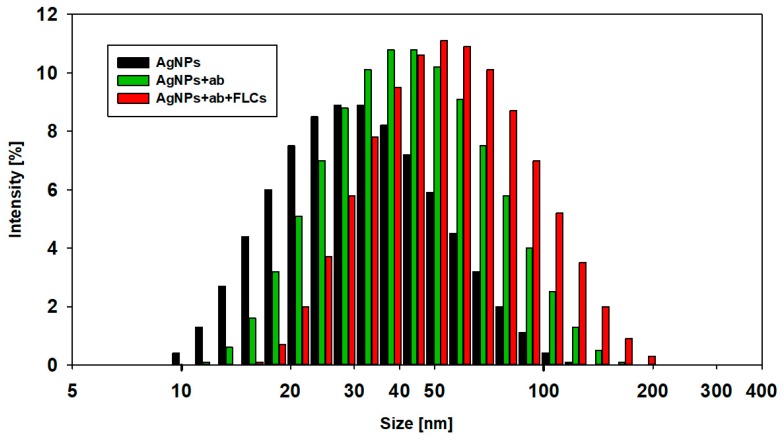
Dynamic light scattering size distribution (by intensity) curves for AgNPs (black), AgNPs + ab (green), and AgNPs + ab + FLCs (red) solutions.

Prepared AgNPs were characterized using TEM, UV/Vis spectrophotometry (Figure 2 and Figure 3), and DLS measurement (Figure 4). The UV/Vis spectrum shows that the resulting AgNPs solution has a characteristic absorption maximum at 395 nm. As the maximum of the plasmon resonance spectrum and diameter of AgNPs are correlated, a maximum of 395 nm indicates the occurrence of nanoparticles, with dimensions of around 10 nm, which is in accordance with the literature data [18].

The data were confirmed by direct determination of AgNPs’ diameter, obtained from TEM images. The mean TEM-based diameter of AgNPs was 9.62 nm (median 9.2 nm, SD 3.4 nm). Figure 3 shows the TEM particle size distribution of AgNPs.

Dynamic light scattering analysis was performed in order to evaluate the physical characterization of silver nanoparticles (Figure 4). The mean diameter of AgNPs, calculated by intensity distribution, was 30 ± 6 nm. The mean and median values of the diameter of the AgNPs, obtained from DLS, were higher than those obtained from TEM. This may be explained by the interference with the hydrodynamic diameter of AgNPs caused by the dispersant. The DLS method measures the mean hydrodynamic diameter, which is heavily weighted toward the largest structures in the solution, while TEM shows particles’ diameter without this aqueous and ionic cargo. Thus, DLS showed a larger mean particle size. Similar observations were described in the study of Souza et al. [19]. As the particle size distribution is not narrow, the presence of bigger particles may contribute to an increase in light scattering, which increases the size of the measured particles.

### 3.2. Preparation of ab-Modified AgNPs for the Detection of FLCs 

**Figure 5:**
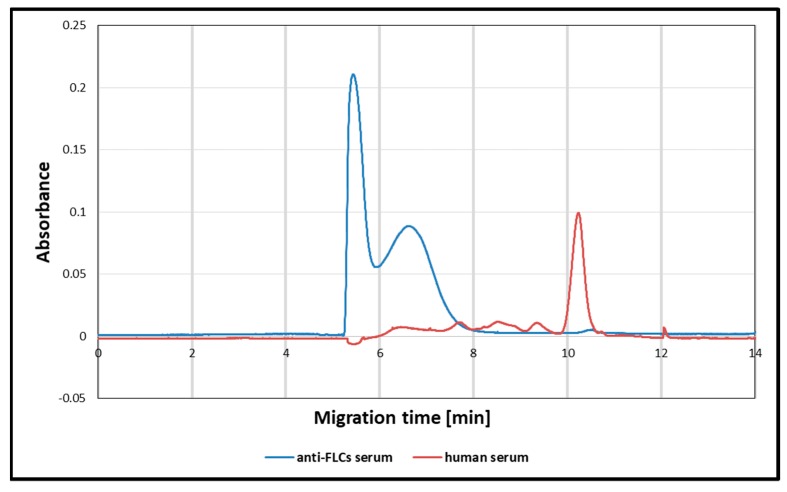
Capillary electropherogram of the anti-FLCs serum (blue line) and human serum, as a reference (red line).

For the detection of protein antigens, either mono- or polyclonal antibodies can be used. In the case of immunoglobulin free light chains, which display significant heterogeneity, it is possible that the monoclonal antibody will not detect a particular epitope. However, it is difficult to characterize the commercially available polyclonal antibodies. From a technical point of view, polyclonal antibodies can consist of a whole serum or purified immunoglobulin fraction. To characterize the composition and the concentration of the antibodies used in this study, capillary electrophoresis was performed (Figure 5). The electrophoretic pattern demonstrated that the anti-FLCs serum was composed of a purified gamma globulin fraction. The concentration of the immunoglobulin stock solution, estimated through a comparison with the known concentration of a protein standard (using the capillary electrophoretic method), was 168 g/L. This corresponds to 1.2 mM, assuming a molecular immunoglobulin IgG weight of 140 kDa. As the silver nanoparticles were coated with antibodies by adding 1 μL of the anti-FLCs antiserum (stock solution) to 10 mL of the AgNPs solution, the final concentration of antibodies was 120 nM.

One of the most important steps in designing an immunochemical test is to determine the absolute concentration of all elements and the optimal antibody/antigen molar ratio in order to exclude the possibility of an antigen excess (the hook effect).

The next problem that must be solved is the determination of the antibody/nanoparticle ratio affecting protein corona formation. This is a complicated process that depends on the electrostatic and hydrophobic interactions between specific antibodies and a nanoparticle’s surface [9,20].

Literature data on the optimal number of antibodies that should be used for the functionalization of nanoparticles are very divergent. There are huge discrepancies concerning the optimal number of antibodies that should be added. Sometimes, the data even differ by an order of magnitude. Additionally, the authors present different concentrations of nanoparticles in solutions and numbers of added antibodies. Some authors present the concentration of AgNPs in milligrams of elementary silver/liters [12,15,21].

To clarify the interpretation of the results obtained in this study, all of the concentration data were presented in mols/liters. This approach provides information concerning AgNPs/antibodies/antigen ratios.

In this study, the molar concentration of the solution of AgNPs was estimated, on the basis of the LSPR maximum and the molar extinction coefficient (5.56 × 10^8^ M^−1^ cm^−1^), to be 10 nM. The concentration of antibodies, after the functionalization process, was 120 nM. This means that the AgNPs/antibodies ratio was about 10. With this number of antibodies, the nanoparticles are stable, and they do not aggregate spontaneously. 

**Figure 6:**
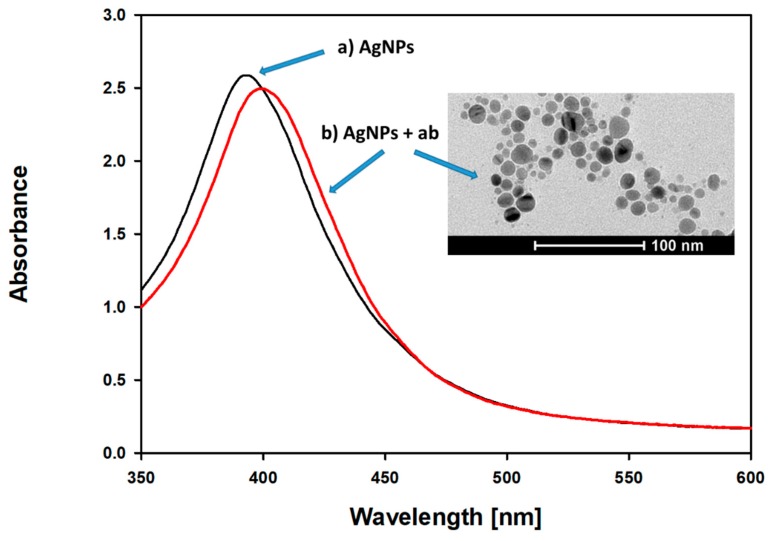
UV-Vis absorption spectra and TEM image: (**a**) silver nanoparticles (AgNPs) and (**b**) silver nanoparticles after coating with antibodies (AgNPs + ab). The TEM image highlights the protein corona surrounding the individual grains of nanoparticles.

To characterize the antibody functionalization process, UV-Vis LSPR spectra were recorded. Compared to citrate-stabilized AgNPs, antibody functionalization results in a slight red shift and a decrease in the absorption maximum, without peak broadening, which is in agreement with previous studies [22] (Figure 6). The LSPR spectrum right shift indicates the formation of a protein corona.

The DLS measurement shows that the mean diameter of the AgNPs-ab, calculated by intensity distribution, is 46.1 ± 4.1 nm (Figure 4). The typical dimensions, reported in the literature, of immunoglobulins are approximately 14.5 nm × 8.5 nm × 4 nm [23]. The presented DLS data suggest that immunoglobulins formed a monolayer on the surface of AgNPs during the functionalization step. Additionally, the binding site of the immunoglobulins on the silver nanoparticle surface seems to be in the constant Fc domain, leaving the two antigen-recognition Fab regions available for antigen binding. 

The zeta potential of the modified AgNPs is higher than that of the citrate-stabilized AgNPs (−27.3 mV vs. −36.6 mV), and theoretically, they should be more prone to aggregation. While the Zeta potential increased, the antibody-coated AgNPs were stable in the solution. This confirms previous observations that the coverage of nanoparticles with proteins increases their stability. Due to protein–nanoparticle hydrophobic and ionic interactions, protein-coated nanoparticles do not aggregate [24].

### 3.3. Detection of FLCs Using ab-Functionalized AgNPs 

**Figure 7:**
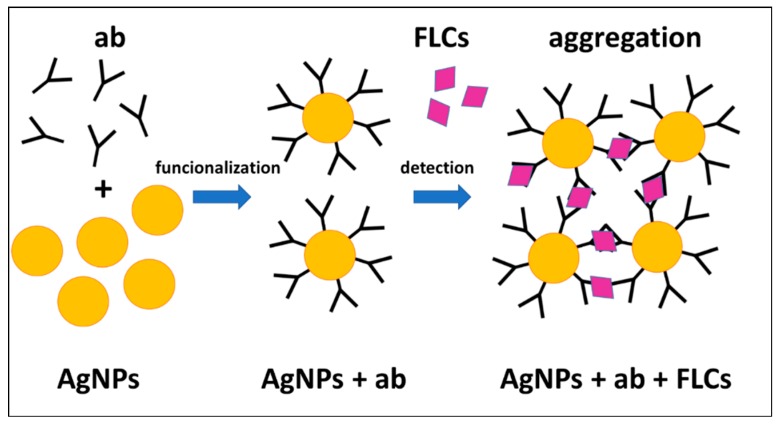
The mechanism of antigen detection. Specific antibodies were attached to the citrate-stabilized AgNPs. A further addition of the protein antigens (FLCs) caused the nanoparticles to aggregate.

The detection of FLCs in the probe is based on the interaction between specific antibody-functionalized AgNPs and neoplastic cell-derived protein antigens (FLCs) (Figure 7).

The addition of immunoglobulin free light chains to ab-functionalized AgNPs is accompanied by spectacular changes in the absorption spectra related to LSPR. These spectral changes are related to the aggregation process of AgNPs, which can be observed by TEM, DLS measurements, and direct observations using laser light scattering.

**Figure 8:**
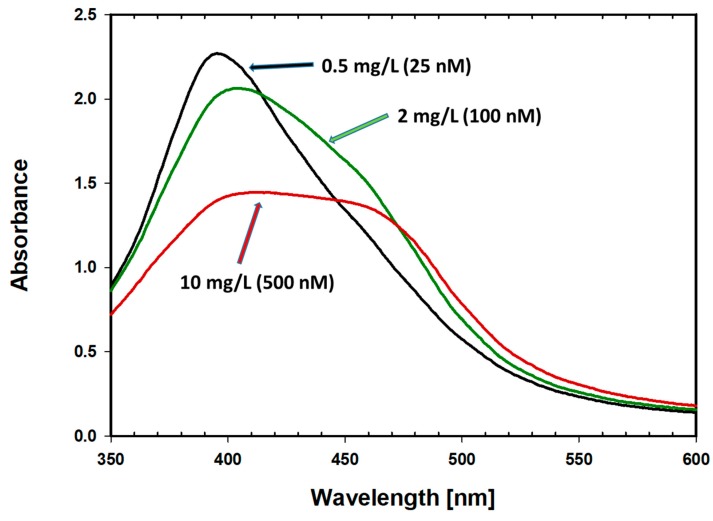
Spectral changes of the nanoparticle aggregates after the addition of different numbers of immunoglobulin free light chains to antibody-coated nanoparticles [10 nM]. The final FLCs concentrations were within a range of 0.5–10 mg/L (25–500 nM).

Increasing concentrations of FLCs are accompanied by a decrease in the maximum absorption at 395 nm, broadening, and red-shift of the LSPR spectrum. Additionally, the formation of a second peak, at a wavelength of around 470 nm, was observed. Spectral changes that occur after the addition of different numbers of immunoglobulin free light chains to antibody-coated nanoparticles (with 24 h of incubation) are presented in Figure 8.

Concentrations of FLCs (0.5–10 mg/L [25–500 nM]) in the presence of antibody-functionalized AgNPs results in a decrease in absorbance at 395 nm. Further increasing the FLCs concentrations results in regrowth of the absorbance and subsequent narrowing of the absorbance peak.

**Figure 9:**
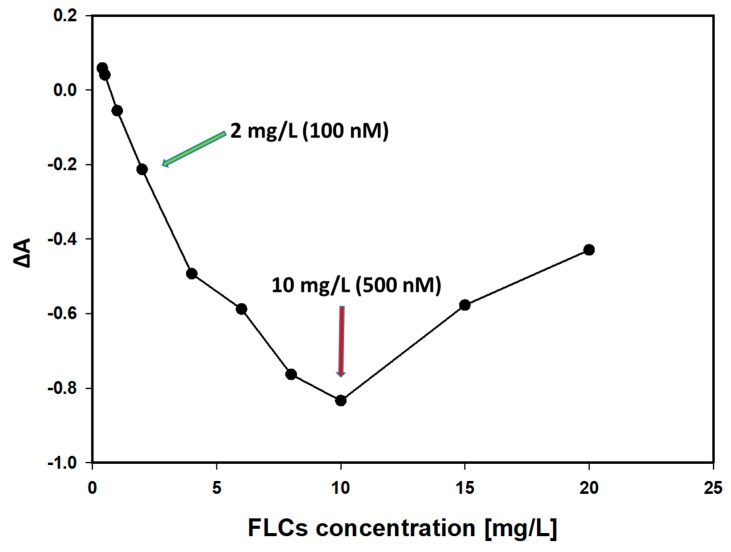
Changes in the absorbance of antibody-coated nanoparticles (10 nM), with the maximum LSPR (395 nm) and increasing FLCs concentrations. The biggest changes were observed at a FLCs concentration of 10 mg/L (500 nM).

Figure 9 shows the changes in the absorbance (at 395 nm) of antibody-coated AgNPs (10 nM) with increasing FLCs concentrations. The maximal absorbance changes were obtained when the FLCs/AgNPs molar ratio was 50. Further increasing the FLCs concentrations results in a regrowth of the absorbance. This can be explained on the basis of the antibody–antigen hook effect.

**Figure 10:**
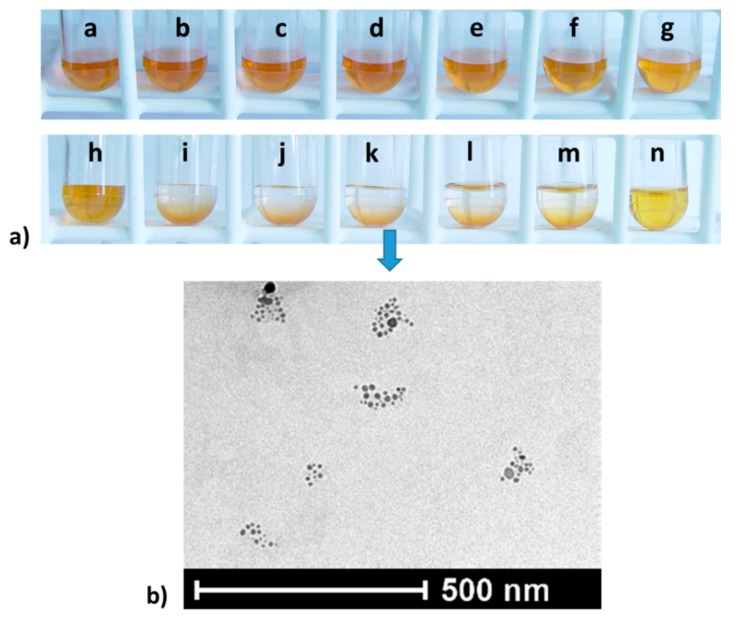
(**a**) Color changes of the AgNPs + ab + FLCs solutions, with FLCs concentrations from 0 to 22 mg/L, which are represented by the letters a–n. (**b**) TEM image of silver nanoparticle aggregates (k). Changes in the absorbance of antibody-coated nanoparticles (10 nM), with the maximum LSPR (395 nm) and increasing FLCs concentrations. The biggest changes were observed at a FLCs concentration of 10 mg/L (500 nM).

Spectral changes were accompanied by a change in the color of the solution. When the samples were centrifuged, sedimentation of the AgNPs in the samples, with the optimal concentration ranges, took place (Figure 10).

The largest spectral changes occurred at FLCs concentrations of up to 10 mg/L. At higher concentrations (above 20 mg/L), the reaction was blocked, most likely by a protein corona that forms as a consequence of excess protein (antigen).

The range of concentrations at which the reaction may occur indicates the minimal clinically useful sensitivity. This silver NP-based assay suffers from typical immunochemical characteristics, resulting from the antigen excess, i.e., the so-called hook effect. In practice, as in other immunochemical tests, the only way to solve this problem is by diluting the sample at higher antigen concentrations [25].

### 3.4. Detection of FLCs Using ab-Functionalized AgNPs 

**Figure 11:**
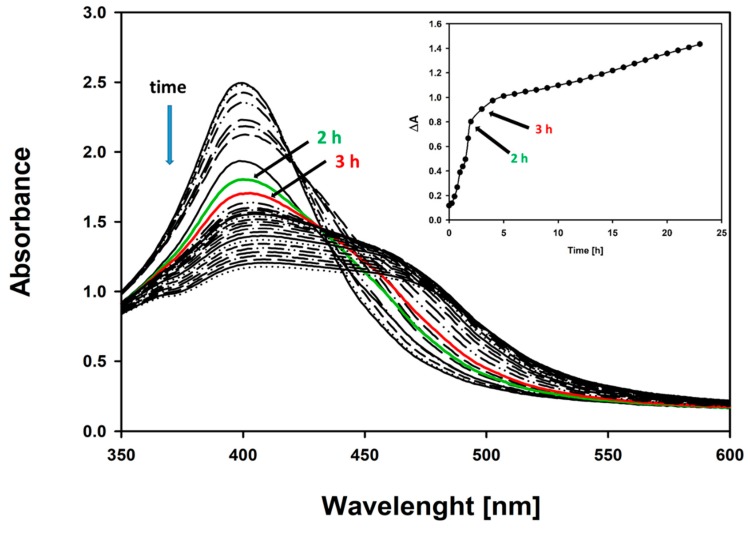
Kinetics of the immunological reaction, with the selected number of FLCs (7 mg/L). Characteristic changes in the absorption spectrum: up to the 2nd h, a decrease in the maximum absorbance at 395 nm occurred; and later, a widening of the spectrum towards longer wavelengths (red-shift) and the formation of a second peak at 470 nm was observed. The inset presents time-dependent absorbance changes at 395 nm. The biggest absorbance changes were observed during the first 2 h of the reaction.

To further characterize the FLCs measurement assay using AgNPs, the kinetics of the reaction was measured at the selected FLCs concentration: 7 mg/L (300 nM) (Figure 11).

The biggest changes in the LSPR spectra occur within the first 2 h (Figure 11 inset). The immunochemical reaction was divided into two phases. During the first 2 h, a decrease in the maximum absorption at 395 nm was observed. Then, in the second phase, the LSPR absorbance peak broadens, and a red-shift and second peak at a wavelength of around 470 nm appears (Figure 11) [26]. 

This aggregate formation-induced red-shift in the nanoparticle LSPR resonance spectra has also been well documented in relation to other metal colloids. Similar changes in the absorbance spectra during the reaction between the antibody-functionalized gold NPs were described in the clinically important immunochemical assay for the detection of glycated hemoglobin [27].

This aggregation behavior, found by UV/Vis spectroscopy, was confirmed by DLS measurements of the hydrodynamic diameters and zeta potential of antibody-functionalized AgNPs. As the immune reaction progressed, an increase in the hydrodynamic diameter of the objects and an increase in the zeta potential were observed.

**Figure 12:**
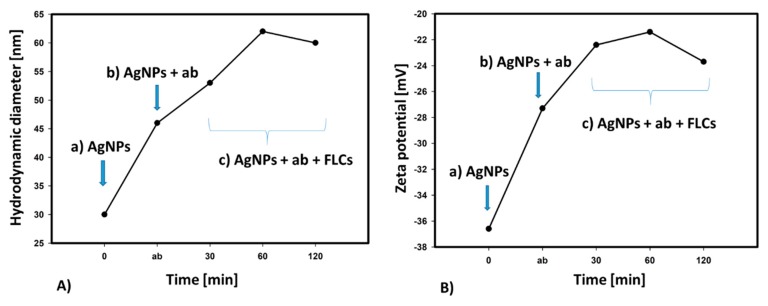
Changes in the hydrodynamic diameter (**A**) and zeta potential (**B**) of (**a**) the citrate-stabilized AgNPs (AgNPs), (**b**) antibody-functionalized AgNPs (AgNPs + ab) and (**c**) silver nanoparticles coated with antibodies, after the addition of FLCs (AgNPs + ab + FLCs).

As the immune reaction progresses and protein particles of the measured antigens (FLCs) selectively interact with specific antibodies, the hydrodynamic diameter and zeta potential of the aggregates increase (measured 30, 60, and 120 min from the beginning of the reaction). An increase in the zeta potential promotes the aggregation of ab-coated AgNPs (Figure 12). After 60 min, the second stage of the reaction is visible, as indicated by the decrease in the hydrodynamic diameter and zeta potential of the aggregating nanoparticles, which is consistent with the formation of a distinct set of absorbance spectra (Figure 11).

### 3.5. Investigation of Antibody-Functionalized Nanoparticle Aggregation by Direct Observation Using Laser Light Scattering

The aggregation of antibody-coated silver nanoparticles was also examined by analyzing the scattering of monochromatic green (532 nm) semiconductor laser light. In the measurement system constructed for the purposes of the present research, various patterns of the light scattering of uncoated nanoparticles, nanoparticles coated with antibodies, and silver nanoparticle–protein aggregates were demonstrated.

**Figure 13:**
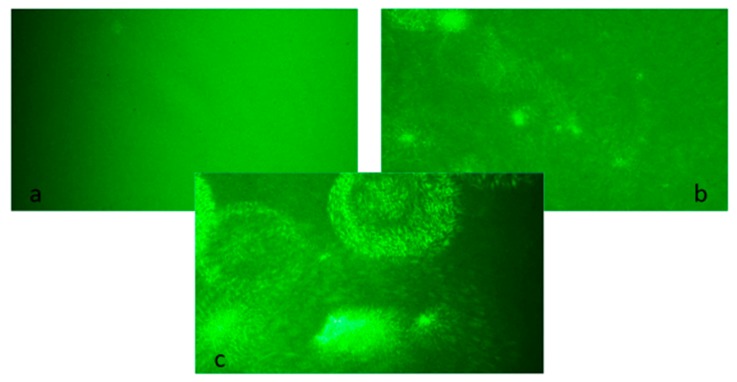
Scattering patterns of monochromatic green semiconductor laser light, passing through: (**a**) citrate-stabilized silver nanoparticles (AgNPs), (**b**) antibody-functionalized silver nanoparticles (AgNPs + ab), and (**c**) nanoparticle aggregates (AgNPs + ab + FLCs).

A narrow beam of laser light, passing through the solution of unfunctionalized silver nanoparticles, gave a homogenous scattering pattern (Figure 13). The functionalization of the nanoparticles with antibodies caused the formation of small light scattering clusters. The addition of protein antigens (FLCs) resulted in the formation of intense light scattering aggregates, which can easily be recognized by analyzing the light dispersion patterns. The obtained scattering patterns were consistent with the results obtained by transmission electron microscopy and sedimentation observations. The data from the light scattering measurements may be used for the qualitative real-time investigation of the protein integration process. The light scattering patterns correlate with the diameter of AgNPs, which can be further quantitated by image analysis software. This method has the potential to offer high sensitivity, enabling the observation of the aggregation of single nanoparticles [28]. The results obtained by DLS demonstrated an increased mean hydrodynamic diameter of nanoparticles (62.8 nm vs. 30 nm for citrate-stabilized AgNPs). However, the DLS method does not show the formation of discrete clusters.

The standard methods used for monitoring the immunoglobulin-/antigen-related aggregation of nanoparticles—UV/VIS spectrophotometry, TEM imaging, and dynamic light scattering (DLS)— generally provide consistent results. However, each of these methods has some limitations, which are due to the method used for preparing a sample for the study or the research methodology itself, among other factors. Different scattering patterns visible in the microscope observations coincide with the results obtained by other methods.

The pattern scattering method can be a cheap, relatively simple research method for studying protein interactions using silver nanoparticles.

During the creation of protein corona aggregation research systems, a lot of elements related to the physical and chemical properties of nanoparticles and protein ligands should be considered.

When choosing appropriate imaging methods, nanoparticle-based technology may be useful for tracking other reactions related to protein aggregation, e.g., in the study of amyloidosis and other diseases associated with the formation of protein deposits.

## 4. Conclusions

This paper provides an example of the use of silver nanoparticles in the study of protein interactions. The study was focused on a specific, disease-related protein antigen. The obtained model can be practically used to construct an immunochemical test, with a high diagnostic potential.

During the development of the method, an appropriate method of synthesizing and functionalizing nanoparticles should be selected to obtain structures with the desired physicochemical properties.

The effect of the immunological reaction between silver nanoparticles coated with anti-lambda free light chain antibodies and the target antigen, free light chains of immunoglobulins, were absorbance changes in the area of the maximum surface plasmon resonance, depending on the FLCs concentration. In another nanomolar concentration range of FLCs (25–500 nM; 0.5–10 mg/L), changes in absorbance were accompanied by the aggregation of nanostructures. This is the range of clinically useful concentrations, applicable in the diagnosis of monoclonal gammopathy. It is therefore possible to use the created system to construct a diagnostic test for the quantitative determination of free light chains of immunoglobulins. Similar tests have a key application in the diagnosis and monitoring of monoclonal gammopathy. It is also expected that this novel method can be used in the detection of other serum or urine proteins.

Different methods for studying the aggregation of nanoparticles provide consistent, method-specific, and complementary results. The method based on laser light scattering, which was developed in this study, enables the direct observation of the aggregation of single nanoparticles in real time. It combines all of the observations obtained by other methods: UV-Vis spectrophotometry, transmission electron microscopy, and dynamic light scattering. It can be a relatively simple qualitative research method for studying the protein interactions in biological systems using silver nanoparticles.
